# MINT and IntAct contribute to the Second BioCreative challenge: serving the text-mining community with high quality molecular interaction data

**DOI:** 10.1186/gb-2008-9-s2-s5

**Published:** 2008-09-01

**Authors:** Andrew Chatr-aryamontri, Samuel Kerrien, Jyoti Khadake, Sandra Orchard, Arnaud Ceol, Luana Licata, Luisa Castagnoli, Stefano Costa, Cathy Derow, Rachael Huntley, Bruno Aranda, Catherine Leroy, Dave Thorneycroft, Rolf Apweiler, Gianni Cesareni, Henning Hermjakob

**Affiliations:** 1 Department of Biology, University of Rome, Tor Vergata, Via della Ricerca Scientifica, 00133 Rome Italy; 2 EMBL - European Bioinformatics Institute, Wellcome Trust Genome Campus, Cambridge, CB10 1SD, UK

## Abstract

**Background:**

In the absence of consolidated pipelines to archive biological data electronically, information dispersed in the literature must be captured by manual annotation. Unfortunately, manual annotation is time consuming and the coverage of published interaction data is therefore far from complete. The use of text-mining tools to identify relevant publications and to assist in the initial information extraction could help to improve the efficiency of the curation process and, as a consequence, the database coverage of data available in the literature. The 2006 BioCreative competition was aimed at evaluating text-mining procedures in comparison with manual annotation of protein-protein interactions.

**Results:**

To aid the BioCreative protein-protein interaction task, IntAct and MINT (Molecular INTeraction) provided both the training and the test datasets. Data from both databases are comparable because they were curated according to the same standards. During the manual curation process, the major cause of data loss in mining the articles for information was ambiguity in the mapping of the gene names to stable UniProtKB database identifiers. It was also observed that most of the information about interactions was contained only within the full-text of the publication; hence, text mining of protein-protein interaction data will require the analysis of the full-text of the articles and cannot be restricted to the abstract.

**Conclusion:**

The development of text-mining tools to extract protein-protein interaction information may increase the literature coverage achieved by manual curation. To support the text-mining community, databases will highlight those sentences within the articles that describe the interactions. These will supply data-miners with a high quality dataset for algorithm development. Furthermore, the dictionary of terms created by the BioCreative competitors could enrich the synonym list of the PSI-MI (Proteomics Standards Initiative-Molecular Interactions) controlled vocabulary, which is used by both databases to annotate their data content.

## Background

Molecular interactions are the heart of cellular physiology, and protein-protein interactions specifically play a key role in a multitude of cellular functions, from signal transduction to gene expression regulation. Thus, knowledge of the interaction networks of cells is fundamental to understanding the roles played by each protein in the cellular machinery. The recent development of high-throughput methodologies for the study of protein-protein interactions offers great promise for the compilation of the cellular interactomes. The volume of data thus generated requires the development of informatics tools for storing, querying and analyzing the data.

The molecular interaction databases MINT (Molecular INTeraction) [1,2] and IntAct [3,4] were conceived for the purpose of storing experimentally verified protein-protein interactions reported in peer-reviewed journals. Not all experimental methods and experimental setups are equally trustworthy. For instance, some techniques, although useful for mapping the interaction domains, are performed *in vitro*, and therefore in the absence of cellular factors that may modulate the interaction; whereas for *in vivo *techniques the system is often perturbed in order to facilitate the detection of an interaction. Both MINT and IntAct therefore endeavor to capture a full representation of the interaction data available in the literature to allow users to determine the reliability of an interaction. With the aim of achieving complete literature coverage, the two databases (along with other major public interaction data providers) founded the International Molecular Exchange Consortium (IMEx) [5] for sharing curation efforts and for exchanging completed records on molecular interaction data.

**Figure 1:**
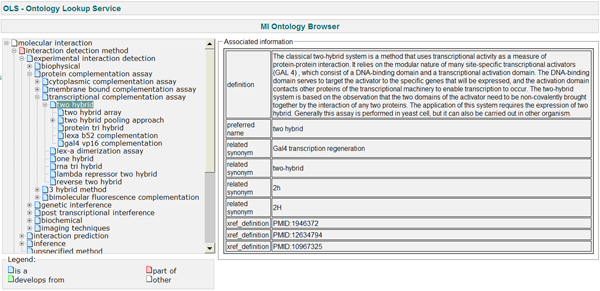
An overview of the PSI-MI CV in OLS. CV, controlled vocabulary; MI, Molecular Interactions; OLS, Ontology Lookup Service; PSI, Proteomics Standards Initiative.

One of the most important recent advances in interaction data annotation is the development of the PSI-MI controlled vocabulary (CV) [6]. This was developed by the Molecular Interactions (PSI-MI) work group of the Human Proteome Organization Proteomics Standards Initiative (HUPO-PSI) [7] and consists of a standardized and hierarchical ontology of terms used for accurately describing interaction data. The PSI-MI CV terms provide an in-depth description of the term to which the various synonyms used in literature can be mapped. Thus, the PSI-MI CV greatly aids consistent, unambiguous annotation and is a boon to data exchange in several respects. First, it permits annotators to describe interaction data fully without resorting to free text; this makes annotation faster and less error prone. Second, when applied in accordance with the agreed standards, the CV permits seamless exchange of data between databases, because no mapping from one lexicon (or one set of semantic rules) to another is required. For instance, to describe an experiment in which a GST-tagged molecule is over-expressed in a eukaryotic cell, pulled down with affinity beads, and interacting partners identified by mass spectrometry, curators can describe the experiment with the most appropriate CV terms available. In the absence of a shared CV, databases may employ free text descriptions that can vary between individual curators and databases, or have separate in-house CVs that do not map to each other. Thus, IntAct and MINT curate data using the PSI-MI CV terms in order to describe interaction data consistently. Advances in experimental techniques for determining and characterizing interactions are reflected in the continual evolution of the CV. A snapshot of the hierarchical PSI-MI CV is shown in Figure 1. The data itself is stored and disseminated in the PSI-MI 2.5 standard, an XML exchange format [8].

Deposition into public databases is a mandatory prerequisite for publication of nucleic acid sequences, protein sequences, and protein structures. However, this is not yet the case for molecular interaction data; journals are only now starting to make such database submission mandatory [9]. Nevertheless, also the upload of high-throughput experiments data requires a curation effort. Thus, the efficient extraction of molecular interaction data from already-published literature is necessary to populate the publicly available databases. Furthermore, in the case of high-throughput experiments the only way to upload the information is through a manual curation of the data usually supplied as supplementary materials. To date, the only reliable way to achieve this is through manual curation, which is a time-consuming and laborious process. The development of effective text-mining tools could complement manual curation by speeding up the information extraction process, thus permitting increased literature coverage. For instance, text mining tools could facilitate the mapping of protein interactors to their UniProtKB [10] identifiers, as well as selecting the text that best describes the interaction and matching this text to appropriate PSI-MI CV terms. However, for a full and accurate description of interactions, a manual element is still required (see Challenges for automatic extraction, below).

The BioCreative [11] protein-protein interaction (PPI) task addresses exactly these goals. Competitors were compared and evaluated to determine whose methodologies would most likely be useful in real world scenarios, for instance as an aid to the database curators. To assist with the BioCreative PPI task, IntAct and MINT contributed both a training set for development of algorithms and a test set for objective evaluation of the text-mining tools. Interactions annotated from the test set publications were not publicly released by contributing databases until the BioCreative subtasks were completed. In addition, both databases provided a full description of their curation process, including the paper selection criteria and the quality control processes used to check resulting database records.

## Results and discussion

### Curation standards

**Figure 2:**
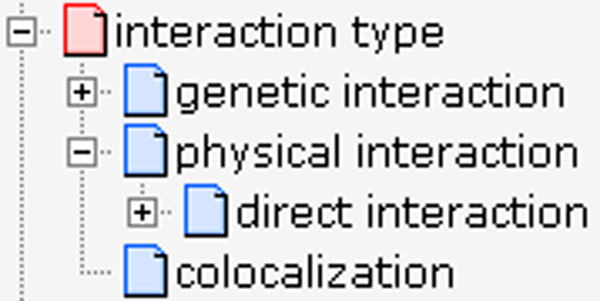
Interaction type in PSI-MI. MI, Molecular Interactions; PSI, Proteomics Standards Initiative.

Syntax and semantics for data representation in MINT and IntAct are provided by the Proteomics Standards Initiative-Molecular Interaction (PSI-MI 2.5) standards, as established by the PSI-MI workgroup, of which MINT and IntAct are core members. This workgroup develops and maintains a common data model for the representation and exchange of interaction data. The schema and the CVs, which allow representation of binary and *n*-nary interactions, are continuously updated to permit increasingly accurate and detailed descriptions of molecular interactions. Interaction records in MINT and IntAct represent either physical interactions or co-localizations (Figure 2) in accordance with the PSI-MI standards, where 'physical interactions' are defined as 'interactions among molecules that can be direct or indirect'. Because genetic interactions describe functional relationship among genes, they are considered distinct from physical interactions between proteins and are not currently curated by MINT and IntAct.

The database curation teams strive to maintain high curation standards. However, a comparison of publications curated by both MINT and IntAct between the years 2003 and 2005 revealed that the two databases annotated exactly the same interaction pairs in only 6 out of 52 publications. The discrepancies were due to partial curation by one or the other database (not all the interactions were annotated), although each publication was meant to be fully curated; most of these partial curations occurred early in the history of MINT and IntAct, when the two databases were still developing their curation standards, including the standards for what constitutes adequate evidence for an interaction. Other discrepancies were due to mapping of an interactor to different isoforms; to incomplete information in the manuscript (the two databases varied in how much additional information they sought from the authors); and occasionally to curation errors. Furthermore, identical PSI-MI CV terms to describe the 'experimental methods' were used by both databases for only nine publications, although terms from the same hierarchical branch had been selected. This suggested that, in many cases, the choice of the method term in the ontology tree is susceptible to curator interpretation so that the same experimental evidence can lead to different interaction records.

Thus, the adoption of the PSI-MI standards *per se *is not sufficient to guarantee identical database records in the different databases. Shared curation rules are also necessary to ensure that the PSI-MI standards are applied consistently between databases. To this aim, as of January 2006 MINT and IntAct have adopted the same curation rules as defined in the IMEx curation manual [12]. This curation manual was optimized with an inter-annotator agreement exercise performed in December 2005 in which the curation of five selected publications performed by the different IMEx members was compared. As a result of the development of common curation rules, no differences were reported in either the identification of the molecules or in the interaction detection methods. This initial small number of publications is currently being expanded as the rules are further developed, ensuring greater curation consistency between databases.

The discrepancies in data supplied by the two databases are not expected to affect the BioCreative PPI subtasks to the same degree. There should have been no impact on the interaction article subtask (IAS), because both databases agreed on which articles contained interaction data. Similarly, the interaction method subtask (IMS) should not have been affected because the databases differed only in the granularity of CV terms used, and the IMS subtask mapped the methods to the root (least granular) terms. The interaction pair subtask (IPS) would have been the most affected because the databases occasionally differed in their identification of interactors. Other discrepancies were in fields not assessed by any subtask. Thus, the discrepancies should have had only minimal impact on the competition; regardless, we recommend using data curated according to IMEx standards in future competitions.

### IntAct/MINT databases contribution to the BioCreative training set

Protein-protein interaction information extracted from articles during the years 2005 and early 2006 formed the contribution of the IntAct and MINT databases to the training dataset. There was no preselection of particular journals within this set. All interactions meeting curation standards were annotated, and the data were made available to the BioCreative organizers in PSI-MI XML2.5 format. Each interaction in the 'training set' has been fully represented, including experimental features such as interaction detection method, participant identification method, post-translational modifications, mutations affecting the interaction, and binding ranges. For the purposes of this competition, the BioCreative participants used only the XML fields reporting the protein identifiers and the experimental detection method.

### IntAct/MINT database contribution to the BioCreative test set

The MINT test set was composed of protein-protein interactions extracted from articles published in *FEBS Letters*, *EMBO Journal*, and *EMBO Reports *between January 2006 and July 2006. The IntAct test set was composed of protein-protein interactions extracted from articles of *Journal of Biological Chemistry *(JBC) and journals belonging to the *Nature *group of publications published in 2006 (Table 1).

**Table 1:** MINT and IntAct contribution to the test-set

	Count of publications	Count of interactions
MINT	221	1,520
IntAct	154	951

Total	375	2,471

As an additional task for the compilation of the BioCreative test set, the curators were asked to identify and report the sentence best describing each interaction from the perusal of either the abstract or the full-text article. In 20% of the cases it was not possible to identify a sentence describing the curated interaction (Table 2).

**Table 2:** Interactions with annotated sentence

	Count of interactions	Count of interactions with sentence	%
MINT	1,520	1,176	77
IntAct	951	801	84

Total	2,471	1,977	80

### Challenges for automatic extraction of protein interaction data by text mining

Here we describe a list of potential problems in the curation process that might affect BioCreative predictions. These are pitfalls that could affect any manual or text mining effort to extract interaction data, potentially leading to inaccurate or incomplete description of information contained in articles.

#### Missing UniProtKB mappings

The major cause of loss of data results from the difficulty in mapping the gene names or identifiers described in the article to UniProtKB entries. This was due to an ambiguous description of the gene name, species, subtype of the protein in question, or more rarely the absence of a UniProtKB entry for the molecule involved in the interaction. A typical case is when authors mention that they used the mammalian protein without specifying whether they are referring to, for example, the mouse or the human form. In other cases the authors mention the use of a multisubunit protein not specifying the subunit. This issue has been recently addressed in the MIMIx (Molecular Interaction Experiment) recommendations [13].

#### Interactions cannot be mined from abstracts

It is not always possible to identify a single sentence that clearly describes an interaction reported in a paper. In many cases the evidence that a paper is eligible for curation is dispersed throughout multiple sentences in the full-text article or may only be in figure or table legends. Nevertheless, curators can clearly identify and extract an interaction from a figure or a table, even if there is no sentence explicitly reporting that interaction in the text. For instance, positive controls are not usually cited in the text and interactions from high-throughput experiments are reported in tables.

#### False positives derived from ambiguous terms

For text-miners the presence of the word 'interaction' in the text directly points to an interaction. Unfortunately, the 'interaction' can refer to experiments describing genetic interactions that are not curated by MINT/IntAct, to drug-drug interactions, or to other data irrelevant to MINT/IntAct. In other cases there is no experimental evidence supporting the interaction that is based only on authors' assumptions. Interactions may also be described based on predictions or model building; these do not constitute physical interactions or co-localization and are not curated by either of the databases.

#### Interactions mediated by complexes

Interactions between protein complexes (for example, Pol II) and proteins are not considered by MINT/IntAct curators. In these cases, the interactions detected by the text-mining tool will not find any match in MINT/IntAct records.

### Contribution to text-mining community

If text mining tools can accurately identify sentences or passages within articles that are indicative of molecular interactions, then they can potentially facilitate manual curation by prescreening the literature. We therefore provided the BioCreative competitors with examples of such sentences and passages.

An annotation topic 'source-text' was introduced in MINT and IntAct. MINT datasets are downloadable from the MINT FTP site [14]. Furthermore, IntAct has continued to extract the interaction sentences; currently, 3,463 sentences are available for 529 publications. IntAct has also introduced an annotation topic 'dataset' with the description 'BioCreative - Critical Assessment of Information Extraction Systems in Biology' to identify the entries that contributed to the BioCreative test set. Both the extracted interaction sentences and the dataset curated for BioCreative competition are available for download from IntAct FTP site [15,16]. The normalized protein interaction sentences generated from the BioCreative initiative were then made available by the organizers for subsequent assessment by database curators.

### Text-mining and the development of the PSI-MI controlled vocabulary

The PSI-MI CV provides a consistent set of terms used to annotate the interaction data. The vocabulary is continuously updated to assimilate newer and more sophisticated techniques. Synonyms, definition, and literature reference for each term are stored within the CV to assist the user in selecting the appropriate term. The dictionary of synonyms developed by the text-mining community, both during the competition and in the future, could be incorporated into the PSI-MI CVs and thereby greatly enhance the CVs.

Manual curation is laborious and it is extremely difficult to quantify the required amount of time to complete the curation of each article; the process of curating a single paper can take up to 1 day of a trained curator's time, much of which is consumed in adding significant value to the interactions. Initial identification of the interactors and interaction technique is followed by an in-depth analysis of the interactors and the interactions. The PSI-MI CV is used extensively to define and describe the interactors and interactions. InterPro signatures [17] and Gene Ontology terms [18] are also used to provide richer interactor annotation to users. The additional steps ensure full and accurate data representation.

IntAct and MINT are currently investigating the possibility of integrating text-mining applications into their curation environment. This is being done at two levels. The first is identifying the publications describing interactions involving a given set of proteins (IAS) [19,20]. The second allows for pre-analysis of full-text publications for unambiguous mapping to UniProtKB entries and identification of the interaction detection method involved (IPS). This analysis can be stored in PSI-MI XML as preliminary data and then be used by a curator to perform the exhaustive annotation of the publication. The results of the full curation are then used to enhance further the tool by indicating which of the predicted interactions were right and wrong. Here again, the PSI-MI XML is used to propagate the feedback to the text-miners.

## Conclusion

MINT and IntAct provide high quality and well documented interaction data from the literature using controlled vocabularies, which reduce the ambiguity in the naming of the techniques and interpretation of interaction features. This is achieved through careful manual curation by highly qualified curators. However, as both the volume of literature and the number of proteins requiring characterization increases, the manual processing capability is soon saturated. Semi-automated assistance would thus greatly expedite the curation process. Text-mining in the biomedical domain is receiving increasing attention. To aid and encourage the development of such tools, the IntAct team at the European Bioinformatics Institute and the MINT team at the University of Rome Tor Vergata agreed to take part in the BioCreative PPI challenge. Both MINT and IntAct contributed to the training set, which can be used to develop the text-mining process, and to the test set, which can be used for the evaluation of the competitors' results.

The interactions themselves are not described in sufficient detail within an article abstract alone, as was demonstrated by the publications that could not be curated from the selected abstracts. This highlights the importance of the full-text (supplementary information included) text-mining process. This is necessary for both the identification of interactors as well as description of the interaction.

At present, manual literature mining can extract more detailed interaction data than is possible by text-mining, and more accurately define the interactors and the interactions. For instance all of the information regarding the mutants could hardly be recovered by text mining because it is dispersed in both the text and the materials and methods. However, to achieve a broad coverage as well as high quality database content, manual curation and text mining can efficiently complement each other.

First of all, the selection of papers of interest for protein-protein interactions could be expedited by adopting tools developed by the IAS. Furthermore, a critical step in literature mining is mapping biological entities to entries in public domain databases such as UniProtKB for proteins. This may require the mapping of highly ambiguous gene/protein names. Automated mapping of the proteins to UniProtKB entries and detection of interactions (IPS) and the extraction of 'interaction detection method' (IMS) from the articles would improve the literature coverage and efficiency of the manual curation process.

A continued interaction between the two communities is necessary to develop an effective text-mining solution to the problems of automated interaction data extraction from published articles.

## Materials and methods

The contributions of the MINT and IntAct databases to the BioCreative PPI task was divided according to the various subtasks of the competition. Curation of entries from PubMed articles was carried out by MINT and IntAct to assist the BioCreative task. The MINT and IntAct data were curated in accordance with to the respective annotation manuals [21,22]. A publication may report one or more experimental methods, each of which may have one or more interactions.

### Determination of the training set

The BioCreative scientific committee determined the training set by selecting articles curated during the years 2005 and early 2006 by the MINT and IntAct databases. For such training set no sentences describing the interaction were available.

### Protein interaction articles subtask IAS: choosing the articles for the test set

An important initial exercise was to select the articles to be curated. This is essential, because not all published articles describe protein-protein interactions. The BioCreative competition committee provided a list of journals available for the curation task.

#### IntAct

Articles were initially chosen from JBC issues released on 6, 13, 20, and 27 January 2006, and 3 and 10 February 2006. This was done by perusing the article abstracts manually and in some cases by rapid reading of the full-text paper looking for interaction information. Forty articles were also curated from other JBC issues or the journals belonging to the *Nature *group of publications. The rest of the articles from these six issues of JBC were classified as not relevant for this task and served as a negative control.

#### MINT

Articles were chosen from issues of *FEBS Letters *(numbers 3, 5, 6, 7, 8, 9, 10, 11, 13, 14, 15, 16, and 17 from 2006), *EMBO Reports *(issues 1, 2, 3, 4, 5, 6, and 7 from 2006), and *EMBO Journal *(issues 1, 2, 3, 5, 6, 7, 8, 9, 10, 11, 12, and 13 from 2006). The rest of the articles from these journals were classified as not relevant for this task and served as a negative control.

The information available within the full-text and supplementary materials of appropriate articles was manually curated into the MINT and IntAct databases.

### Protein interaction pairs subtask IPS: mapping of the interactors to the UniProtKB proteins

The full-text of the article often contained sufficient details to allow the identification of the UniProtKB identifier; where this was not the case, the information in the supplemental materials and/or references sections was used. UniProtKB consists of two sections, UniProtKB/SwissProt and UniProtKB/TrEMBL. The former contains manually annotated records with information extracted from literature and curator-evaluated computational analysis, whereas the latter contains high quality computationally analyzed records enriched with automatic annotation and classification. While mapping to the UniProtKB, a UniProtKB/SwissProt entry was preferentially chosen over a UniProtKB/TrEMBL entry. A TrEMBL entry containing the longest version of the sequence was preferentially used where a choice of only TrEMBL entries was available, because the longer entry is most likely to contain the entire protein sequence. In cases where the paper refers to a protein name that maps to several distinct UniProtKB records, curators used additional information in the paper (such as descriptions of particular residues, for example 'tyrosine 211') to determine the appropriate ID mapping. If the ID cannot be disambiguated in this way, then the interaction is not curated.

The interactor pairs were often determined based on the information available in the figure legends and the results sections of the article.

The IntAct curation team, in the cases where there was no UniProtKB entry and the necessary criteria specified in the annotation manual were satisfied, created a protein entry in the IntAct database. These had only a European Bioinformatics Institute accession number.

### Protein interaction sentences subtask ISS: 'source-text' to describe the interaction

Multiple techniques may describe the interactions between the same two interactors. These techniques and the interactors they detect are described in various places in the article text. The most pertinent text providing information about the interaction detection method and the protein interactors was stored in the MINT and IntAct interaction entries as an annotation using the annotation-topic 'source-text'. Either PDF or HTML forms of the article were used to find the sentences. Many of these protein interaction sentences were taken from the results sections and figure legends of the article. There was no restriction on the number of sentences forming a single 'source-text' description.

### Protein interaction method subtask IMS: mapping of the interaction data to PSI-MI CV

The information about the experimental technique used to determine an interaction was often available in the materials and methods, figure legends, supplemental materials and results sections of the articles. The deepest possible child term of PSI-MI CV root term 'interaction detection method' is used to describe the method in a consistent machine-readable form. Where more than one method in an article identified an interaction, the UniProtKB identifiers for the interactors were reported in the context of all the experimental methods used. Hence, the interaction between the same two interactors may have been described multiple times.

### Assessment of the curation process

The interaction data, entered in the MINT and IntAct databases by the curators as per the respective annotation manuals, was checked using an automated procedure based on predefined curation rules that were designed to detect common errors. These tools detect mandatory fields that have not been filled; furthermore, the IntAct database is currently refining a tool that detects slightly more complex errors, such as an interaction with two baits and no preys. A further evaluation was carried out by a senior curator to ensure that the information in the databases correctly represented the information in the publication. The final data representation was as agreed upon between the senior curator and the primary curator. The authors were notified when the IntAct and MINT records were released, and their examination of the records provided a third level of quality control.

### Release of the test set

All articles curated by IntAct for the BioCreative test set contained the annotation topic 'dataset' with a description 'BioCreative - Critical Assessment of Information Extraction systems in Biology' on the individual experiment. This allowed organizers to download the entire dataset.

## Abbreviations

CV, controlled vocabulary; HUPO, Human Proteome Organization; IAS, interaction article subtask; IMEx, International Molecular Exchange Consortium; IMS, interaction method subtask; IPS, interaction pair subtask; JBC, *Journal of Biological Chemistry*; MI, Molecular Interactions; MINT, Molecular INTeraction; PPI, protein-protein interaction; PSI, Proteomics Standards Initiative.

## Competing interests

The authors declare that they have no competing interests.
